# Krüppel-like Factor 4 Regulates Intestinal Epithelial Cell Morphology and Polarity

**DOI:** 10.1371/journal.pone.0032492

**Published:** 2012-02-24

**Authors:** Tianxin Yu, Xi Chen, Wen Zhang, Juan Li, Ren Xu, Timothy C. Wang, Walden Ai, Chunming Liu

**Affiliations:** 1 Markey Cancer Center, University of Kentucky, Lexington, Kentucky, United States of America; 2 Department of Biological and Molecular Biochemistry, University of Texas Medical Branch, Galveston, Texas, United States of America; 3 Department of Pathology, Microbiology and Immunology, University of South Carolina School of Medicine, Columbia, South Carolina, United States of America; 4 Department of Pharmacology and Toxicology, University of Kentucky, Lexington, Kentucky, United States of America; 5 Division of Digestive and Liver Diseases, Department of Medicine and Irving Cancer Center, Columbia University, New York, New York, United States of America; 6 Department of Molecular and Cellular Biochemistry, University of Kentucky, Lexington, Kentucky, United States of America; University of Birmingham, United Kingdom

## Abstract

Krüppel-like factor 4 (KLF4) is a zinc finger transcription factor that plays a vital role in regulating cell lineage differentiation during development and maintaining epithelial homeostasis in the intestine. In normal intestine, KLF4 is predominantly expressed in the differentiated epithelial cells. It has been identified as a tumor suppressor in colorectal cancer. KLF4 knockout mice demonstrated a decrease in number of goblet cells in the colon, and conditional ablation of KLF4 from the intestinal epithelium led to altered epithelial homeostasis. However, the role of KLF4 in differentiated intestinal cells and colon cancer cells, as well as the mechanism by which it regulates homeostasis and represses tumorigenesis in the intestine is not well understood. In our study, KLF4 was partially depleted in the differentiated intestinal epithelial cells by a tamoxifen-inducible Cre recombinase. We found a significant increase in the number of goblet cells in the KLF4-deleted small intestine, suggesting that KLF4 is not only required for goblet cell differentiation, but also required for maintaining goblet cell numbers through its function in inhibiting cell proliferation. The number and position of Paneth cells also changed. This is consistent with the KLF4 knockout study using villin-Cre [1]. Through immunohistochemistry (IHC) staining and statistical analysis, we found that a stem cell and/or tuft cell marker, DCAMKL1, and a proliferation marker, Ki67, are affected by KLF4 depletion, while an enteroendocrine cell marker, neurotensin (NT), was not affected. In addition, we found KLF4 depletion altered the morphology and polarity of the intestinal epithelial cells. Using a three-dimensional (3D) intestinal epithelial cyst formation assay, we found that KLF4 is essential for cell polarity and crypt-cyst formation in human colon cancer cells. These findings suggest that, as a tumor suppressor in colorectal cancer, KLF4 affects intestinal epithelial cell morphology by regulating proliferation, differentiation and polarity of the cells.

## Introduction

Colorectal cancer is the second most commonly diagnosed cancer among men and women and the second leading cause of cancer deaths in the United States [2], [3]. Different genetic variations could lead to abnormal epithelial development and polyp formation, which could be further induced to progression of colorectal carcinomas [4]. Wnt signaling plays an important role in early stages of colorectal carcinogenesis; abnormality in the gene APC or β-catenin leads to aberrant crypt formation [5], [6]. Mutations in other oncogenes and tumor suppressor genes, such as K-ras and p53, also contribute to colorectal carcinogenesis [4].

KLF4 is a zinc finger transcription factor initially found to be enriched in the epithelium of intestine and skin [7], [8]. Later, it was found in a variety of other tissues, such as thymus, cornea, cardiac myocytes and lymphocytes [9], [10], [11], [12]. KLF4 plays an important role in development and cell differentiation [8], [13], [14]. In normal intestine, KLF4 is predominantly expressed in differentiated epithelial cells near the luminal surface and goblet cells in the crypts [15], [16]. KLF4 is down-regulated in colorectal cancers and has been identified as a tumor suppressor [17], [18], [19]. As one of the four factors that induce pluripotent stem cells, KLF4 plays a role in cell fate reprogramming and self-renewal of embryonic stem (ES) cells [20], [21]. The roles of KLF4 in differentiated intestinal cells are not well understood.

Mice homozygous for a null mutation in KLF4 had defects in terminal differentiation of goblet cells, while further study of KLF4 in mouse intestine was hampered due to early lethality of mutant mice [14]. Using Villin-Cre recombinase system, another study found that conditional ablation of KLF4 from the intestinal epithelium led to failure of goblet cell differentiation [15], which also highlights the role of KLF4 in maintaining intestinal epithelial morphology and homeostasis. Interestingly, depletion of KLF4 from two-week-old mice using vil-CreER, an inducible Cre recombinase, had no effect on goblet cell differentiation [22]. The discrepancy may be due to differential expression of the villin gene in early and later stages of gut development [23]. In this study, we analyzed the role of KLF4 in the adult intestine using an inducible Cre recombinase, which is driven by native promoter of KLF4.

## Results

### KLF4 loss leads to change in number of goblet cells and morphology of the small intestinal epithelium

**Figure 1:**
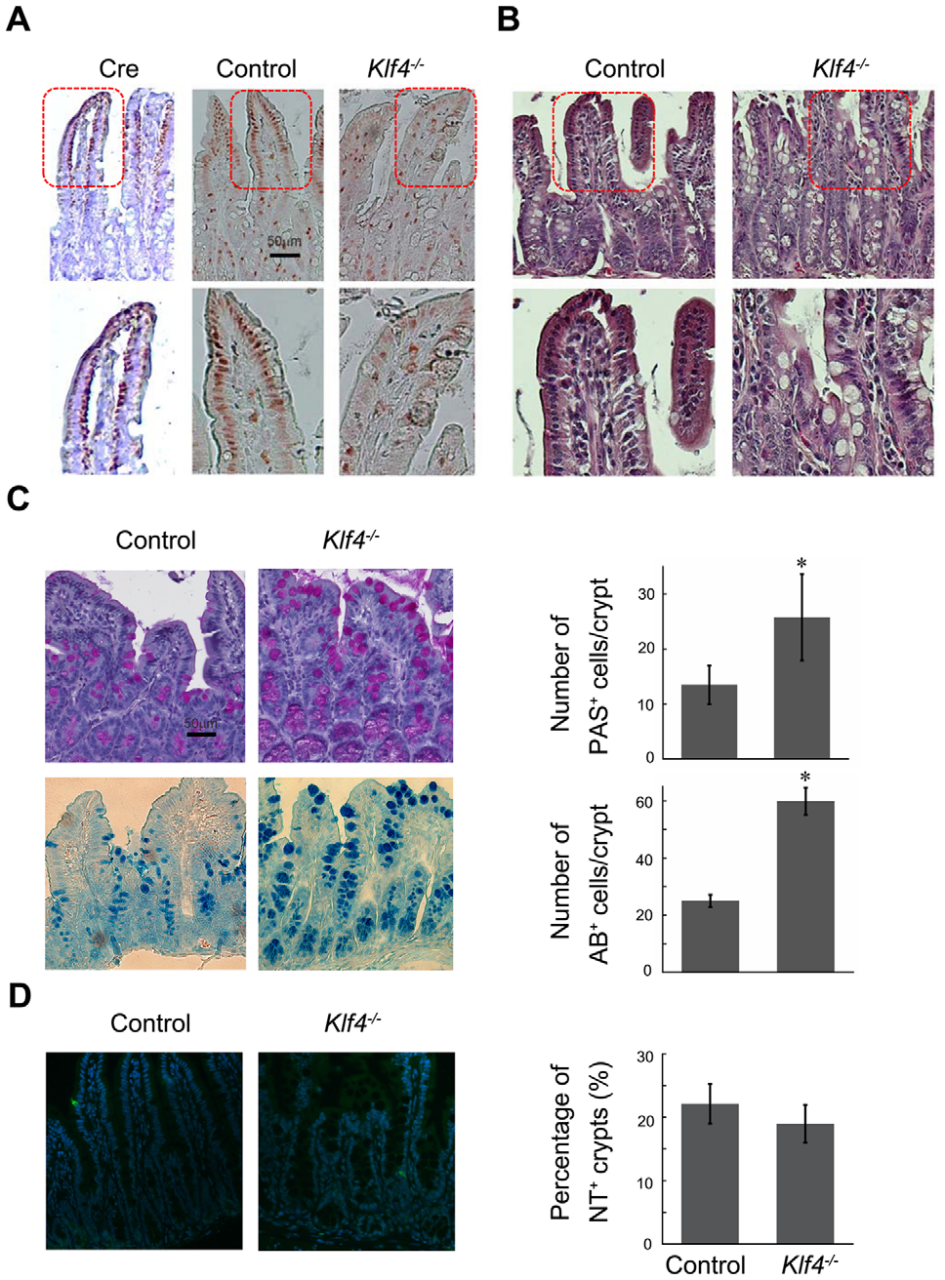
KLF4 loss leads to change in number of goblet cells and morphology of the small intestinal epithelium. (A) Left: IHC staining for Cre recombinase in *Klf4^−/−^* small intestine. Right: IHC staining for KLF4 in control and *Klf4^−/−^* small intestine tissues. (B) H&E staining of control and *Klf4^−/−^* small intestine tissues. (C) Small intestine treated with tamoxifen for 5 days were stained for Periodic acid-Schiff (PAS, top) and Alcian Blue (AB, bottom). (*, P<0.05) (D) Tissue slides from small intestine of control and *Klf4^−/−^* mice were stained for neurotensin (NT) antibody and detected by immunofluorescent antibody.

In order to test the function of KLF4 in adult intestinal epithelium cells, we generated inducible KLF4 knockout (*Klf4* ^−/−^) mice, which are KLF4/CreER (+/−) and KLF4(flox/flox) double transgenic. The Cre recombinase cDNA fused with tamoxifen-inducible estrogen receptor gene was inserted into BAC clone at the initiating methionine of KLF4 gene. Thus, the expression of Cre recombinase is driven by the KLF4 promoter in transgenic mice. Induction of KLF4/CreER (+/−) and KLF4(flox/flox) double transgene with tamoxifen led to activation of Cre recombinase. The KLF4 function in the skin was studied using this mouse model. KLF4 depletion resulted in a significant increase of hair follicle density, as well as changes of suprabasal cells from a single layer into multiple layers, which is indicating an inhibitory role of KLF4 in proliferation of mouse skin keratinocytes [24]. In the small intestine, the Cre recombinase was predominantly expressed in the top of the villus, and which is recapitulating expression pattern of endogenous KLF4 (Fig. 1A). Tamoxifen-mediated Cre recombinase activation resulted in partial depletion of KLF4 when compared with non-induced transgenic mice (Fig. 1A).

**Figure 2:**
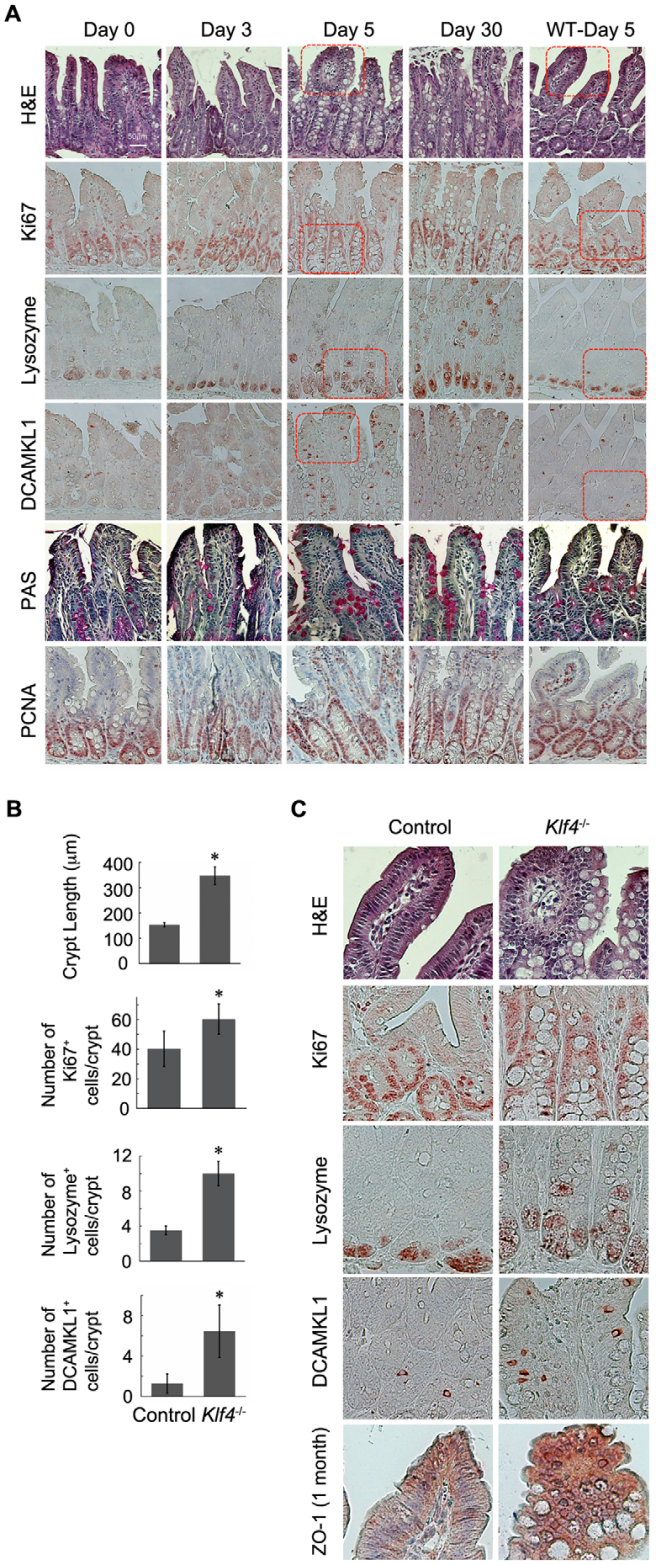
KLF4 ablation leads to abnormal proliferation and differentiation in small intestinal epithelium. (A) Small intestine from *Klf4^−/−^* mice induced by tamoxifen for different time endurances were stained by H&E and PAS, and also immunohistochemistry staining was performed with anti-Ki67, anti-Lysozyme, anti-DCAMKL-1, and anti-PCNA antibodies respectively. (B) Statistic analysis of IHC staining results from (A). (*, P<0.05) (C) IHC staining from (A) in higher magnification of highlighted frames. Bottom panel: IHC staining with ZO-1 antibody in one-month knockout intestine tissue.

Haematoxylin and eosin (H&E) staining results indicated an increase in the number of secretory cells in *Klf4^−/−^* intestine; the position of these cells appeared to be dislocated compared with control intestine (Fig. 1B). To analyze the effects of KLF4 depletion on goblet cells, which are one of the secretory cell lineages in the small intestine, tissue sections were stained with both Periodic acid-Schiff (PAS) and Alcian Blue (AB), respectively (Fig. 1C left panel). An enlargement in size and an increase in the numbers of PAS and AB positive cells indicated an increase in goblet cell proliferation in small intestine of *Klf4* ^−/−^ mice (Fig. 1C right panel), which highlights the role of KLF4 in maintaining numbers of goblet cells in mature small intestine. Time point-specific changes in number of PAS positive cells due to tamoxifen treatment further indicated that KLF4 is critical for goblet cell number maintenance (Fig. 2A). It is worth noticing that our result is distinct from the finding that KLF4 knockout leads to loss of Goblet cells in the colon [14], and that conditional ablation of KLF4 also leads to loss of goblet cells in the intestinal epithelium [15]. The difference is due to the stage of KLF4 knockout before or after goblet cell differentiation. KLF4 depletion had no effect on neuroendocrine cells, as indicated by immunofluorescent staining for neurotensin (NT) (Fig. 1D), suggesting that function of KLF4 in small intestine is cell type-specific.

### KLF4 ablation leads to abnormal proliferation and differentiation in small intestinal epithelium

In order to further examine the role of KLF4 in intestinal epithelial cells, the morphology change was analyzed in *Klf4* ^−/−^ mice compared with non-induced (Day 0) mice by H&E staining (Fig. 2A): The average length of the crypt-villus axis was increased in *Klf4* ^−/−^ mouse intestine (Fig. 2A, 2B). The number of secretory-like cells is increased; these cells either have larger volume of vacuoles or contain secreted granules like Paneth cells (Fig. 2A). A large number of cell nuclei lost apical-basolateral polarity, which is typical of the wild-type enterocytes. Instead of a monolayer of well-oriented epithelial cells, *Klf4* ^−/−^ intestine had multiple layers of disorganized cells (Fig. 2A, C). Positions of the secretory cells were changed; instead of sitting at the bottom of the crypt, the granule-containing cells dislocated upward in the crypts (Fig. 2A, C). In order to confirm that the morphology change was not due to tamoxifen treatment, small intestine from wild-type (WT) mice treated with tamoxifen was stained as a control; they showed a normal morphology as non-treated transgenic mice.

The cell proliferation marker Ki67 was analyzed by IHC. The average length of Ki67^+^ region along the crypt-villus axis, as well as numbers of Ki67^+^ cells increased (Fig. 2A–C), i.e., proliferation compartment of the intestine was expanded, indicating an increase in proliferation capacity in *Klf4* ^−/−^ mouse intestine. PCNA is another proliferation marker; and its change in response to KLF4 loss is consistent with the results from Ki67 staining (Fig. 2A). This further highlights the role of KLF4 in inhibiting intestine proliferation.

In addition to goblet cell staining, the role of KLF4 in intestinal cell proliferation was confirmed by staining for other cell types including Paneth cells and tuft cells. Tissue slides from both normal and *Klf4* ^−/−^ intestine were stained for lysozyme, which is a marker for Paneth cells (Fig. 2A and C). A larger proportion of cells stained positive for lysozyme in small intestine from *Klf4* ^−/−^ mice compared with control mice, and these cells were dislocated through the crypt-villus axis, indicating that KLF4 loss also led to an increase in Paneth cell population and has an effect on position of these cells. This result re-emphasizes the role of KLF4 in controlling Paneth cells and strongly supports the finding from the KLF4 knockout study using villin Cre [15].

Based on the current model, small intestine is composed of the Paneth cell region (bottom of crypt), the stem cell zone (through +4 location), an amplification compartment (up to top of crypt) and a differentiation compartment (including crypt-villus junction) [25], [26]. To further analyze the effect of KLF4 on intestinal homeostasis, tissue sections from *Klf4* ^−/−^ mouse intestine were stained for stem cell and/or tuft cell marker DCAMKL-1 [27] (Fig. 2A–C). Surprisingly, DCAMKL-1 positive cells were increased in the *Klf4* ^−/−^ mouse small intestine along the villus, but were not restricted to the crypt base, indicating an increase in number of tuft cells due to loss of KLF4.

Based on our observation of changes in cell position as well as epithelial apical-basolateral morphology, we proposed that KLF4 is not only responsible for controlling cell differentiation and proliferation, but also cell polarity. As indicated by H&E staining, a great part of the cell nuclei lost polarity in *Klf4^−/−^* mouse small intestine. Paneth cells change their position due to depletion of KLF4. Meanwhile, most of the tuft cells that were positive for DCAMKL-1 also were out of direction in knockout mice (Fig. 2A, C). In order to investigate the role of KLF4 in regulating cell polarity, we generated a three-dimensional (3D) epithelial cyst formation assay for Caco-2 cells, in which the morphological structure of cyst and apical-basolateral cell polarity can be examined *in vitro*.

### KLF4 is essential for cell polarity and crypt-cyst formation in 3D culture of Caco-2 cells

**Figure 3:**
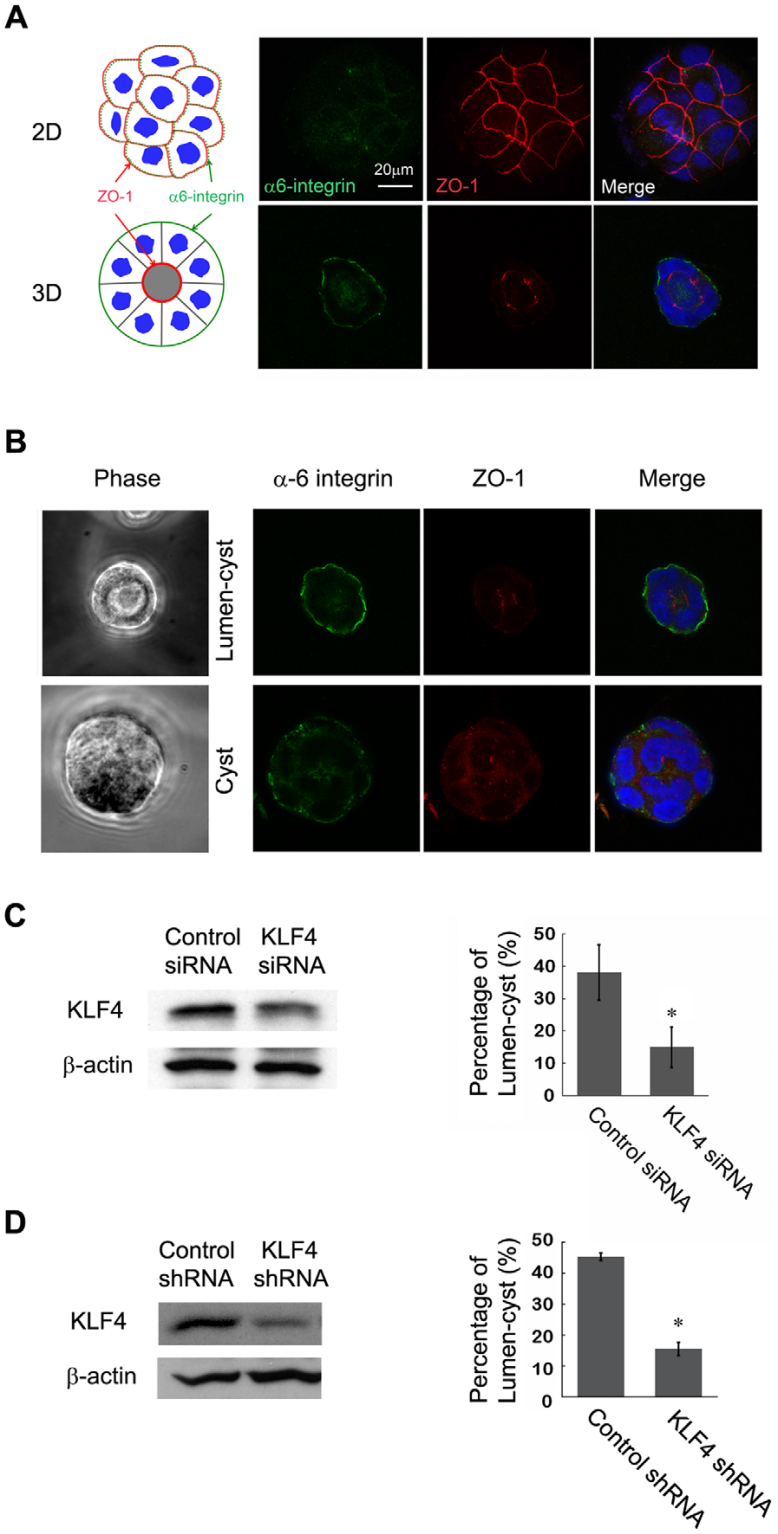
KLF4 is essential for cell polarity and crypt-cyst formation in 3D culture of Caco-2 cells. (A) Immunofluorescent staining of Caco-2 cells in 2D and 3D culture with anti-α6-integrin and ZO-1 antibodies. (B) Caco-2 cells in 3D culture were stained for differentiation markers indicating cell polarity and cyst formation (definition of lumen-cyst vesus cyst structures). (C) Left: western blotting showing knockdown of KLF4 in Caco-2 cells. Right: statistical analysis of percentage of lumen-cyst formation in Caco-2 cell 3D cultures, comparing between control and KLF4 siRNA-transfected cell cultures. (*, P<0.05) (D) Left: western blotting showing expression of KLF4 in 293T cells co-transfected with human KLF4 and KLF4 shRNA plasmids. Right: statistical analysis of percentage of lumen-cyst formation in Caco-2 cell 3D cultures, comparing between control and KLF4 shRNA-infected cell cultures. (*, P<0.05).

Normally, Caco-2 cells with high polarity form lumen-containing cysts in matrigel-based 3D culture and show apical-basolateral polarity as indicated by ZO-1 as a basolateral marker and α6-integrin as an apical marker, respectively (Fig. 3A, bottom; Fig. 3B, top); low- or no-polarity Caco-2 cells only form cysts without lumen (Fig. 3B, bottom). Staining of α6-integrin and ZO-1 for Caco-2 cells in 2D culture is shown as control, indicating non-polarization of Caco-2 cells in 2D growth conditions (Fig. 3A, top). To determine the role of KLF4 in lumen-cyst formation, KLF4 was depleted in Caco-2 cells by siRNA and shRNA delivery approaches, respectively (Fig. 3C and 3D left panels), followed by 3D formation assay. The number of lumen-cysts and total number of cysts were counted and the percentage of lumen-cyst was calculated to indicate the measure of cell polarity. We found that the efficiency of lumen-cyst formation was significantly reduced by siRNA and shRNA (Fig. 3C and 3D), suggesting that KLF4 is essential for cell polarity formation in the 3D culture of Caco-2 cells. To examine the role of KLF4 in apical-basolateral polarity in the intestine, we stained the knockout intestine tissues with ZO-1 antibody and found that KLF4 does regulate ZO-1 expression and distribution in the intestinal epithelial cells: instead of being highly expressed in the outer layer of epithelial cells surrounding the villus, KLF4 knockout intestine had overexpressed ZO-1 in multiple layers of villus (Figure 2C). This confirmed that KLF4 does not only regulate polarity formation of Caco-2 cells, it also regulates apical-basolateral polarity in intestinal epithelial cells.

### KLF4 facilitates cell polarity and crypt-cyst formation in colon cancer cells

**Figure 4:**
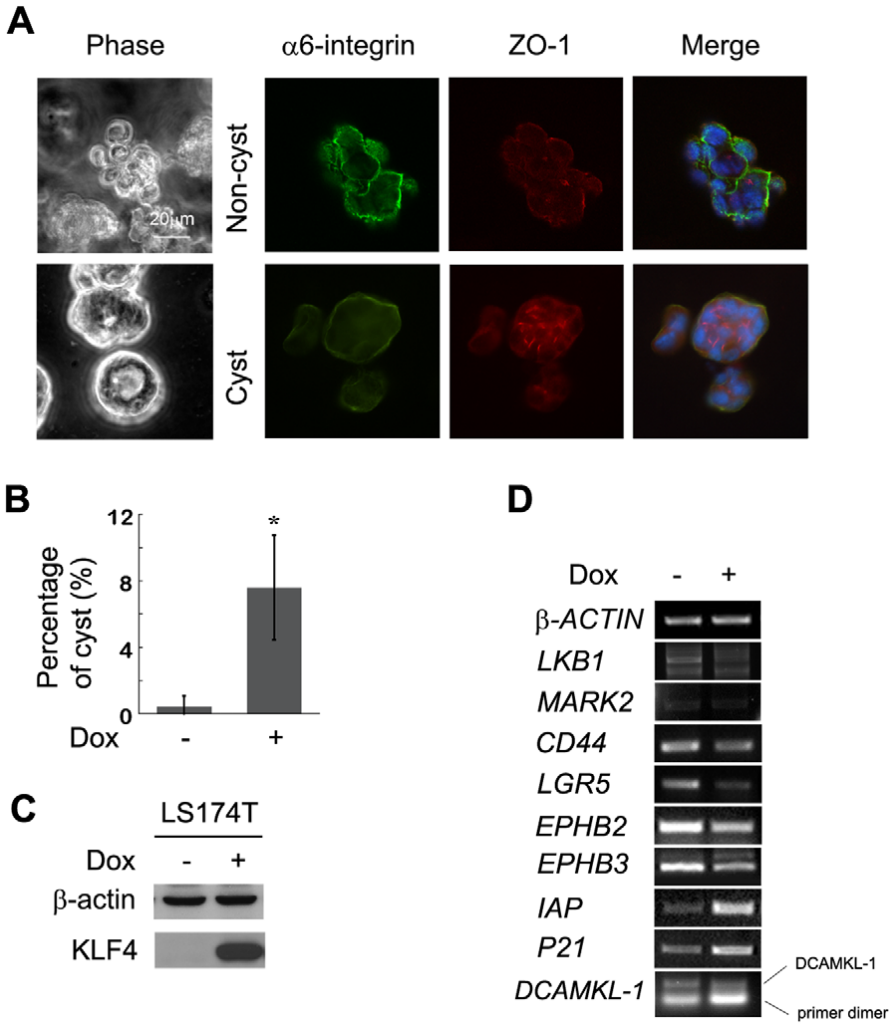
KLF4 facilitates cell polarity and crypt-cyst formation in colon cancer cells. (A) LS174 cells in 3D culture system were stained for differentiation markers indicating cell polarity and cyst formation. (B) Statistical analysis of percentage of cyst formation in LS174T cell 3D cultures, comparing between doxycycline (Dox)-induced and non-induced cells.(*, P<0.05) (C) Western blotting indicating expression of KLF4 in LS174T cell line with or without induction of doxycycline. (D) Semi-quantitative RT-PCR showing expression of genes related to KLF4-regulated cell polarity and related to Wnt signaling.

In order to confirm the role of KLF4 in facilitating cell polarity formation, 3D culture assay was performed in another colon cancer cell line to test whether KLF4 can enhance cyst formation *in vitro*. The LS174T-KLF4 stable cell line expresses KLF4 upon doxycycline induction [18]. LS174T cells seldom form cysts, even under 3D culture conditions. However, induction of KLF4 expression in LS174T cells significantly increased the chances of cyst formation in 3D culture (Fig. 4A, B), indicating that KLF4 indeed enhances cell polarity and thus facilitates cyst formation in 3D formation assay.

In order to address the mechanism by which KLF4 regulates cell polarity both in a knockout mouse model and in a 3D culture system, a panel of cell fate and polarity-related genes were analyzed by semi-quantitative RT-PCR. KLF4 was induced by doxycycline in LS174T-KLF4 colon cancer cells (Fig. 4C). Though we didn't see significant changes in DCAMKL-1 transcription, several polarity-related genes, *LKB1*, *EPHB2*, and *EPHB3*, were down-regulated. Intestinal stem cell markers *LGR5* and *CD44* were also down-regulated. As controls, the differentiation marker *IAP* and cell cycle inhibitor *P21*, which are known KLF4 target genes, were up-regulated by KLF4 (Fig. 4D). These findings suggest that KLF4 regulates epithelial cell polarity by regulating the transcription of multiple genes.

## Discussion

As an important regulator in intestinal cell differentiation during early development, KLF4 is also essential in maintaining normal homeostasis and morphology in adult intestine. Previous studies have deleted KLF4 in embryonic stages of mouse intestine; the terminal differentiation of goblet cells was decreased in these mice [14], [15]. Here, we reported that in mature mouse intestine, partial depletion of KLF4 resulted in an increase in the number of goblet cells, indicating that KLF4 is required not only for goblet cell differentiation in early stages, but also for maintaining the number of differentiated goblet cells, probably by inhibiting cell proliferation. This is consistent with the observation that KLF4 is strongly expressed in goblet cells [15], [16], [19]. We found that the average length of crypts was increased in KLF4-depleted small intestine, and the number of Ki67 positive cells was also increased. In agreement with previous findings, the number and position of Paneth cells had also changed [15]. DCAMKL-1 is a marker for tuft cells, and a potential marker for quiescent intestinal stem cells [28], [29]. We found that the number and position of DCAMKL-1 positive cells was also altered by KLF4 depletion. The changes in morphology and polarity of intestinal epithelial cells were confirmed by H&E staining. These data suggest that KLF4 plays a key role in maintaining normal intestinal homeostasis and morphology by regulating cell differentiation, proliferation and polarity. The roles of KLF4 in cell polarity were further analyzed in 3D culture, and several novel KLF4 target genes involved in cell differentiation and polarity were identified.

Our results suggest tamoxifen-induced knockout of KLF4 is advantageous in tissue- and stage-specificity. We were able to partially deplete KLF4 in the villi of small intestine, where KLF4 normally predominantly expresses. In addition, the inducible knockout strategy allows normal development of small intestine in the early stage of development, which assures that lineage differentiation (i.e., the ability to differentiate Paneth and goblet cells) and intestine function is not affected by KLF4 depletion. The limitation of our model is that KLF4 depletion is not complete. KLF4 is more efficiently deleted in differentiation cells, but less efficiently deleted in progenitor cells that have low levels of KLF4. The daughter cells differentiated from these progenitor cells may express high levels of KLF4. Thus, this mouse model can be used to study KLF4 function in differentiated cells, but is not suitable to study KLF4 function in cell fate determination during stem cell differentiation.

As to the function of KLF4 in cell proliferation, KLF4 plays a crucial role in maintaining the integrity of the cell cycle [30]. Low levels of KLF4 mRNA are essential for cell proliferation [7]. In our study, the proliferating compartment of the intestine in *Klf4* ^−/−^ mice was increased while the total length of the villus-crypt axis turned out to be increased as well, suggesting the role of KLF4 in inhibiting outgrowth of the intestine villus-crypt beyond normal length. The numbers of goblet cells, Paneth cells and tuft cells were increased in KLF4 depleted small intestine, further suggesting that KLF4 inhibits proliferation of certain cell types and thus contributes to maintaining normal cell populations in the intestine. KLF4 also regulates the proliferation of stem cells and/or tuft cells, as indicated by DCAMKL-1 staining (Fig. 2). In control mouse intestine, DCAMKL-1 positive cells were mainly located in the stem cell zone and amplification compartment; in *Klf4* ^−/−^ mouse intestine, the number of DCAMKL-1 positive cells increased significantly in both the amplification and differentiation compartments. DCAMKL-1 has been suggested to be a marker for gastrointestinal stem cells and adenoma stem cells [28], [29]. However, others suggest that DCAMKL-1 only identifies tuft cells since they are not always located at the stem cell position, nor do they co-express with markers of any of the main lineages constituting the intestinal epithelium [27]. The identity of DCAMKL-1 positive cells and the potential roles of KLF4 in intestinal stem cells remain to be determined.

Our previous work demonstrated that KLF4 crosstalks with Wnt signaling in the intestine [16], [18]. Wnt signaling induces maturation of Paneth cells [31] and mediates cell positioning in the intestinal epithelium [32]. The abnormal numbers and locations of Paneth cells could be partially due to enhanced Wnt signaling as a result of KLF4 depletion. The role of Wnt signaling in goblet cell is not clear. Goblet cell numbers were decreased by either activation of Wnt signaling through APC deletion or inhibition of Wnt signaling by DKK1 overexpression [33], [34]. Notch signaling also regulates goblet cells [22]. It is possible that the differentiation and proliferation of goblet cells are regulated by multiple signaling pathways and different developmental stages.

Based on the observation of changes in cell position and apical-basolateral polarity in *Klf4^−/−^* intestine epithelia, together with results from the 3D intestinal epithelial cyst formation assay, we demonstrated that KLF4 regulates intestinal epithelial cell polarity in addition to cell differentiation and proliferation, thus affecting morphology and homeostasis of the intestine.

Several genes that regulated cell polarity were repressed by KLF4, including *LKB1*. As a ‘master’ regulator of cell polarity, LKB1 was reported to induce complete polarity in intestinal epithelial cells; depletion of *LKB1* in Caco-2 cells led to impairment of spontaneous polarization [35], [36]. Recently, it was reported that that CDX2 deficiency leads to abnormal apical-basal polarity in intestinal epithelial cells [37] and that CDX2 deficiency leads to elevated expression of LKB1 [38]. Since KLF4 expression is dependent on CDX2 in human colon cancer cells [39], our finding is consistent with these reports and suggests that KLF4 regulates cell polarity through multiple genes, including *LKB1*.

In summary, the results from this study and previous studies suggest that KLF4 has multiple functions. In the early embryonic stage, KLF4 induces goblet cell differentiation in intestinal epithelium; throughout intestinal development, KLF4 maintains homeostasis of normal intestinal growth and keeps epithelial cells from over-proliferation. Meanwhile, KLF4 regulates apical-basolateral polarity of the intestinal epithelial cells. After all, the intestinal homeostasis and morphology are regulated by multiple factors, including KLF4 and its target genes.

## Materials and Methods

### Transgenic mice and animal work

#### Ethics Statement

Mouse experiments were performed under the approval by the Institutional Biosafety Committee (IBC) and by the Institutional Animal Care and Use Committees (IACUC) of University of South Carolina (Proposal number 1573).

Transgenic mice were generated using a Cre recombinase derived from a bacterial artificial chromosome (BAC, RP23-322L22) containing mouse KLF4 gene [40]. A Cre recombinase cDNA was fused with estrogen receptor gene and was inserted into KLF4 locus at the initiating codon, and the CreER gene transcription is under the control of KLF4 promoter. KLF4 knockout in KLF4/CreER (+/−)/KLF4 (flox/flox) double transgenic mice was induced by 100 mg/kg tamoxifen intraperitoneally (i.p.) for 5 consecutive days at 4 weeks old. Expression of KLF4 as well as multiple genes in wild-type (*Klf4^+/+^*) and knockout (*Klf4^−/−^*) mice was analyzed 3, 5 or 30 days after induction by immunohistochemistry (IHC) staining of fixed intestine tissues.

### Cell culture and 3D formation assay

Caco-2 human colonic epithelial cell line [41] was cultured in high glucose Dulbecco's modified Eagle's medium (DMEM), supplemented with 10% fetal bovine serum and 1% penicillin/streptomycin. For 3D culture, approximately 1.5×10^5^ cells were embedded into 250 µl of 80–90% matrigel. The 3D matrix was allowed to harden in a 24-well plate at 37°C for 30 minutes, then 500 µM of DMEM medium with 2% fetal bovine serum was added and cysts were allowed to form over 5–7 days at 37°C.

LS174T colon cancer cell line [18] was grown in RPMI medium (Mediatech) supplemented with 5% fetal bovine serum and 1% penicillin/streptomycin. Stable cell line LS174T-tet/on-KLF4 has been described previously (Zhang et al., 2006). LS174T-tet/on-KLF4 cells were plated at approximately 2×10^5^ cells per well in a 6-well plate. The following day, doxycycline (1 µg/ml) was added to the culture medium. After 24 h of incubation, cells were trypsinized and counted, then followed by 3D formation assay as indicated with Caco-2 cells.

### Western Blotting

Cells were lysed in the appropriate volume of lysis buffer (50 mM HEPES, 100 mM NaCl, 2 mM EDTA, 1% glycerol, 50 mM NaF, 1 mM Na_3_VO_4_, 1% Triton X-100, with protease inhibitors). The following antibodies were used: mouse anti-β-Actin (Sigma, A1978), mouse anti-Flag (Sigma, F1804).

### RT-PCR

LS174T-tet/on-KLF4 cells were plated at approximately 2×10^5^ cells per well in a 6-well plate. The following day, doxycycline (1 µg/ml) was added to the culture medium. After 48 h of incubation, RNA was isolated using the RNeasy kit (Qiagen). Reverse transcriptase PCR (RT-PCR) was performed as described previously (Zhang et al., 2006). The following primers were used: β-actin, 5′-CAACCGCGAGAAGATGAC-3′ and 5′-AGGAAGGCTGGAAGAGTG-3′; *IAP*, 5′-CCATTGCCGTACAGGATGGAC-3′ and 5′-CGCGGCTTCTACCTCTTTGTG-3′; *p21^Cip1/WAF1^*: 5′-CGACTGTGATGCGCTAATGG-3′ and 5′-AGAAGATCAGCCGGCGTTTG-3′; *LGR5*: 5′-CCTGCTTGACTTTGAGGAAGAC-3′ and 5′-ATGTTCACTGCTGCGATGAC-3′; *CD44*: 5′-CAGAATGGCTGATCATCTTG-3′ and 5′-CAAATGCACCATTTCCTGAG-3′; *LKB1*: 5′-GAGGAGGTTACGGCACAAAA-3′ and 5′-CTGTCCAGCATTTCCTGCAT-3′; *MARK2*: 5′-GCCAGAATCAAAAGCAAC and 5′-ATGATGTTTAGTGGGAGG-3′; *BMI1*: 5′-AGCAGAAATGCATCGAACAA-3′ and 5′-CCTAACCAGATGAAGTTGCTG-3′; *EPHB2*: 5′-AAAATTGAGCAGGTGATCGG-3′ and 5′-TCACAGGTGTGCTCTTGGTC-3′; *EPHB3*: 5′-AGCAACCTGGTCTGCAAAGT-3′ and 5′-TCCATAGCTCATGACCTCCC-3′.

### Interference RNA, H&E staining, immunohistochemistry, PAS and AB staining

Interference RNA and immunohistochemistry were tested as described previously (Zhang et al., 2006). Lentiviral stocks were prepared using control shRNA or human KLF4 shRNA on pGIPz vector containing a marker of turbo GFP (Open Biosystems). H&E staining was performed based on standard protocol by Histology Laboratory of the Imaging Facility at University of Kentucky.

For immunohistochemistry staining, the following antibodies were used: KLF4 (Zhang et al., 2006), rabbit anti-human Lysozyme (Diagnostic BioSystems, RP 028-05), rabbit anti-DCAMKL-1 (Abgent, AP7219b), rabbit anti-Ki67 (Novus Biologicals, NB110-89717).

PAS staining was performed based on standard protocol using reagents of PAS Staining System from Sigma (395-B). Alcian Blue (AB) staining was performed according to standard protocol using Alcian Blue 8GX and Fast Red from Sigma (kindly provided by Dr. Tianyan Gao).

### Immunofluorescent staining

Cells grown on cover glass were fixed in 4% paraformaldehyde in PBS at room temperature for 15 min, washed 3 times with PBS, permeabilized with 0.1% Triton X-100 in PBS for 10 min, and then blocked in 5% goat serum in PBS at room temperature for 1 h. Cells were incubated with primary antibodies at room temperature for 2 hours. Antibodies used include rat anti-human CD49f (α6-integrin, BD Pharmingen, 555734) and rabbit anti-ZO-1 (Invitrogen, 61-7300). Then cells were washed 3 times with PBS and further incubated with Alexa-488-labeled anti-rat IgG (1∶500) and Alexa-568-labeled anti-Rabbit IgG (1∶500) diluted in PBS for 40 min. Nuclei were stained by DAPI (Sigma). The cover glasses were washed, mounted on glass slides, viewed and photographed with an Olympus FW1000 confocal microscope.
